# Resolution of signs and symptoms of illnesses among self-medicating undergraduate students of Mbarara University of Science and Technology: a cross-sectional study

**DOI:** 10.1186/s40780-025-00469-8

**Published:** 2025-07-25

**Authors:** Ronald Mushemeza, Hannah Mwebaza, Patience Amutuhaire, Diana Nakwanyi, Silvano Samba Twinomujuni, Silas Ojuka

**Affiliations:** 1 Department of Pharmacy, Faculty of Medicine, Mbarara University of Science and Technology, Mbarara, Uganda; 2 Department of Clinical pharmacy and Pharmacy practice, School of Pharmacy, Kampala International University, Ishaka, Uganda; 3 Department of Pharmacy, Uganda Heart Institute, Kampala, Uganda

**Keywords:** Self-medication, Pattern, Outcome, Association, Resolution of signs and symptoms

## Abstract

**Introduction:**

Self-medication(SM) is highly prevalent among university students in Uganda, This poses various challenges to the local healthcare system and the nation at large. SM is notorious for its undesirable effects like adverse drug events, drug addiction, antimicrobial resistance, progression of disease to more complicated forms, prolonged morbidity and death. However, some studies have documented reports of resolution of signs and symptoms among self-medicating individuals. This study purposed to investigate the association between patterns of SM and resolution of signs and symptoms among undergraduate students enrolled at Mbarara University of Science and Technology (MUST).

**Methods:**

A descriptive cross-sectional study was conducted at Mbarara University of Science and Technology in Uganda from February 2024 to April 2024. A physical close-ended self-administered questionnaire was used to collect data from respondents. Analysis was done using SPSS version 25. The patterns of SM and the proportion of respondents who reported resolution of signs and symptoms were analysed and presented using descriptive statistics. Pearson’s Chi-square was performed to analyse the association between patterns of SM and resolution of signs and symptoms at the significance level of *P* < 0.05.

**Results:**

Out of 387 respondents, the prevalence of self medication was 71.1% (275/387). Majority of respondents who self-medicated, 85.1% (234/275), reported resolution of signs and symptoms of their illnesses. Most respondents who self-medicated were treating cough, 65.1% (179/275), and headache, 58.5% (161/275). Most of them used cough medications, 63.3% (174/275), and over-the-counter pain relievers, 51.3% (141/275). In the current study there was a statistically significant association between resolution of signs and symptoms and; having cough, (X^2^(1, *N* = 178) = 3.851, *p* = 0.050), having sore throat, (X^2^(1, *N* = 64) = 4.983, *p* = 0.026), and use of cough or cold remedies, (X^2^(1, *N* = 173) = 5.668, *p* = 0.017).

**Conclusion:**

The findings of the current study are suggestive of the popularity of SM among students attending university in sub-Saharan Africa. This study also brings to light the significance of SM to the health care of this population. However, the convenience of resolution of signs and symptoms could mask looming dangers of SM that should be seriously considered by all stakeholders of public health.

**Supplementary Information:**

The online version contains supplementary material available at 10.1186/s40780-025-00469-8.

## Introduction

Self-medication (SM) is a global public health problem that has drawn attention from healthcare systems around the world. There is currently a lack of international consensus on an updated standard definition for SM [1]. However, over two decades ago the World Health Organization(WHO) defined SM as treatment of self-recognized disorders or symptoms by use of medicines without prior consultation by a qualified health professional or intermittent/continued use of medicines previously prescribed by a physician for chronic/recurring diseases [2]. It could involve use of prescription-only medications (POMs) like antibiotics which must be dispensed only after a valid prescription has been presented or over-the-counter (OTC) drugs like paracetamol which are dispensed without requiring a prescription. According to previous research, SM has been observed to be significantly prevalent in institutions of higher education [3–6]. This could be because medications, including POMs, are easily accessible from pharmacies, friends, and coworkers especially in Low- and Middle-Income Countries(LMIC) [7].

The practice of SM involving the use of POMs may be more risky compared to when OTCs are used [8]. Despite the benefit of convenience the risks associated with SM using POMs are numerous including; misdiagnosis, taking the wrong drug dose, using a contraindicated drug, harmful drug-drug interaction, masking of underlying disease [8]. Some of the above risks may also be posed by the use of OTCs. However, POMs are more harzadous due to their unique nature, for instance antibiotics may be affected by antibiotic resistence, opioids can be addictive, drugs like digoxin and warfarin are higly toxic [9]. Unlike the case of POMs, it is legal to purchase medicines allocated as OTCs without a prescription from a valid prescriber [10]. OTCs are also more accessible, affordable, less toxic and more familiar to the community which is possibly why they are less harzadous when used for SM [11].

From a previous study among students attending Mbarara University of Science and Technology(MUST) in south-western Uganda the prevalence of SM was 63.5% [12]. Among undergraduate students of faculty of health sciences at Lira University in northern Uganda a prevalence of 59% was reported [13] whereas the prevalence stood at 69.4% among Kampala university students [14]. These statistics show that SM is a relatively popular practice among university students in Uganda.

The practice of SM is impacted by various factors, for example, in the case of antibiotics; low levels of education and the inaccessibility of appropriate medical care [15]. Unregulated use of both POMs and OTCs may also compound the impact of these factors on SM [16]. Other factors like socioeconomic status of a community may as well influence SM practices among university students. For instance, the prevalence of SM with antibiotics among university students was observed to be more common in Low- and Middle-Income Countries (LMICs) compared to High-Income Countries (HICs) [17].

Theoretically, SM has been extensively documented to be associated with harmful outcomes. Even then, there is a significant scarcity of literature on outcomes of SM among Ugandan communities. Though SM bears a negative connotation, in a few studies it has been associated with some convenient outcomes that may include; time saving, cost saving, resolution of presenting ailments. SM is notorious for negative outcomes that may include but not limited to antimicrobial resistance, prolonged morbidity, drug addiction, drug allergies, habituation, wrong diagnosis or incorrect drug dose [18, 19] and progression of disease to advanced stages [20].

This study explores resolution of signs and symptoms as an outcome among self-medicating undergraduate students enrolled at MUST. It also assesses association between various patterns of SM and resolution of signs and symptoms. Studying this association is necessary because where as some individuals who self medicate may report relief from sickness it is important to investigate if resolution of signs and symptoms occurs randomly or may be associated with certain observed patterns within the community. Otherwise, there is a looming risk of SM being irresponsibly embraced by unsuspecting communities.

## Methodology

### Study aim

To explore association between patterns of SM and resolution of signs and symptoms as an outcome among undergraduate students enrolled at MUST in south-western Uganda.

### Study design

This study adopted a descriptive cross-sectional design; employing quantitative data collection methods.

### Study setting

The current study was conducted at MUST, a public university in south-western Uganda. It was founded in 1989 as a degree-awarding institution in Uganda accredited by the Uganda National Council for Higher Education (NCHE). The Main campus (Kihumuro campus) is approximately 7 km west of Mbarara central business district along the Mbarara -Bushenyi road. The town campus is located in Mbarara city, along the Mbarara-Kabale highway, approximately 269 km south-west of Kampala, Uganda’s capital city.

There are 7368 students at both campuses, of which 6043 are undergraduates and 1325 are postgraduates. The university has five faculties: the faculty of medicine, the faculty of interdisciplinary studies, the faculty of computing and informatics, the faculty of business and management sciences, the faculty of science and the faculty of applied science and technology.

### Study population

All undergraduate students who were enrolled at MUST in the academic year 2023/2024.

### Eligibility criteria

#### Inclusion criteria

- All undergraduate students who were enrolled at MUST in the academic year 2023/2024.

#### Exclusion criteria

- Undergraduate students without a valid MUST identity card.
- Undergraduate students diagnosed with a psychiatric condition.

### Sample size determination

The formula n=[(Zα/2)^2^P(1-p)] ÷ d^2^ (Cochran formula) was used to determine the sample size. The proportion of 63.5% was adopted from a study on SM previously done at MUST [12]. The margin of error was (0.05), 95% confidence level at Z/2(α = 0.05) = 1.96 and design effect = 2. After adding a 10% non-response rate, the final sample was 392.

N (sample size) = *n* + 10% of n.

*n* = 356.155.

*N* = 356.155 + 35.6155.

*N* = 391.7705.

*N* = 392 respondents.

However, out of 392 students who were contacted 387 consented to participate in this study.

### Sampling technique

A stratified random sampling technique was used. Six strata were created from the population. Students were divided into groups based on the faculties that their programs were affiliated with. By choosing respondents at random from each faculty, the sample size was reached based on the percentages of students each faculty contributed to the total student population of the university. A computerized selection of distinct respondents’ university registration numbers without replacement was used to carry out this random selection within each stratum.

### Data collection

A consent form explaining the study background, procedure, and purpose was used to seek informed consent from all the respondents. A physical close-ended self-administered questionnaire was used to obtain all the data from respondents. This tool comprised of three main sections, that is; sociodemographic, self-medication patterns and outcomes of self-medication. The expression, “resolution of signs and symptoms” in the current study meant that the respondents’ signs and symptoms disappeared after SM and did not recur at least up until the time the respondent was participating in the current study. In the tool, data regarding this outcome was elicited using the prompting statement, “symptoms and signs resolved”. After a respondent filled the questionnaire, the filled questionnaire was checked for completeness. The respondent was required to check all boxes that applied to the question. If the respondent checked a box this was considered an affirmative response to the corresponding question while all unchecked boxes were considered negative responses. The collected data was entered into the SPSS software database (version 25) and cleaned. To maintain accuracy of the collected data respondents were asked to fill the questionnaire alone in the absence any friends who might influence their responses. Respondents were not required to indicate any identifiers on the questionnaires. Anonymity was maintained to encourage respondents not to withhold information.

### Data analysis

Data analysis was done using SPSS version 25. The prevalence and patterns of SM were analysed and presented using descriptive statistics. Pearson’s Chi-square was performed to analyse the association between patterns of SM and resolution of signs and symptoms at the significance level of *P* < 0.05.

## Results

### Characteristics of respondents

Out of 392 students, 387 consented to participate in this study, giving a 98.7% response rate. Most of the respondents, 62.8% (243/387), had their ages ranging between 21 and 23 years. Over two thirds, 69.0% (267/387) of the respondents, were males. The vast majority, 95.6% (370/387) of the respondents, were single. Most of the respondents, 71.6% (277/387), resided in hostels/rentals, while majority, 41.6% (161/387), identified as Catholics. About a fifth, 20.7% (80/387) of the respondents, were from the faculty of medicine and over a third, 37.2% (144/387), were in their third year of study. Most respondents, 73.9% (286/387), were privately sponsored students. (Table 1)

**Table 1:** Characteristics of respondents

Variable	Category	Frequency (*n* = 387)	Percentage (%)
Age (years)	18–20	31	8.0
	21–23	243	62.8
	24–26	99	25.6
	27 or above	14	3.6
Gender	Male	267	69.0
	Female	120	31.0
Marital status	Married	15	3.9
	Single	370	95.6
	Divorced	2	0.5
Residence	University halls	74	9.1
	Hostels/rentals	277	71.6
	Commute from home	36	9.3
Religion	Catholic	161	41.6
	Anglican/protestant	138	35.7
	Muslim	32	8.3
	Pentecostal	35	9.0
	Others	21	5.4
Academic sponsorship	Government sponsorship	75	19.4
	Private sponsorship	286	73.9
	Self-sponsorship	26	6.7
Faculty	Faculty of medicine	80	20.7
	Faculty of Interdisciplinary Studies	68	17.6
	Faculty of business and management studies	64	16.5
	Faculty of applied science and technology	62	16.0
	Faculty of computing and informatics	54	13.9
	Faculty of science	59	15.3
Year of study	Year 1	89	23.0
	Year 2	73	18.9
	Year 3	144	37.2
	Year 4	76	19.6
	Year 5	5	1.3

### Prevalence of self-medication

**Fig. 1:**
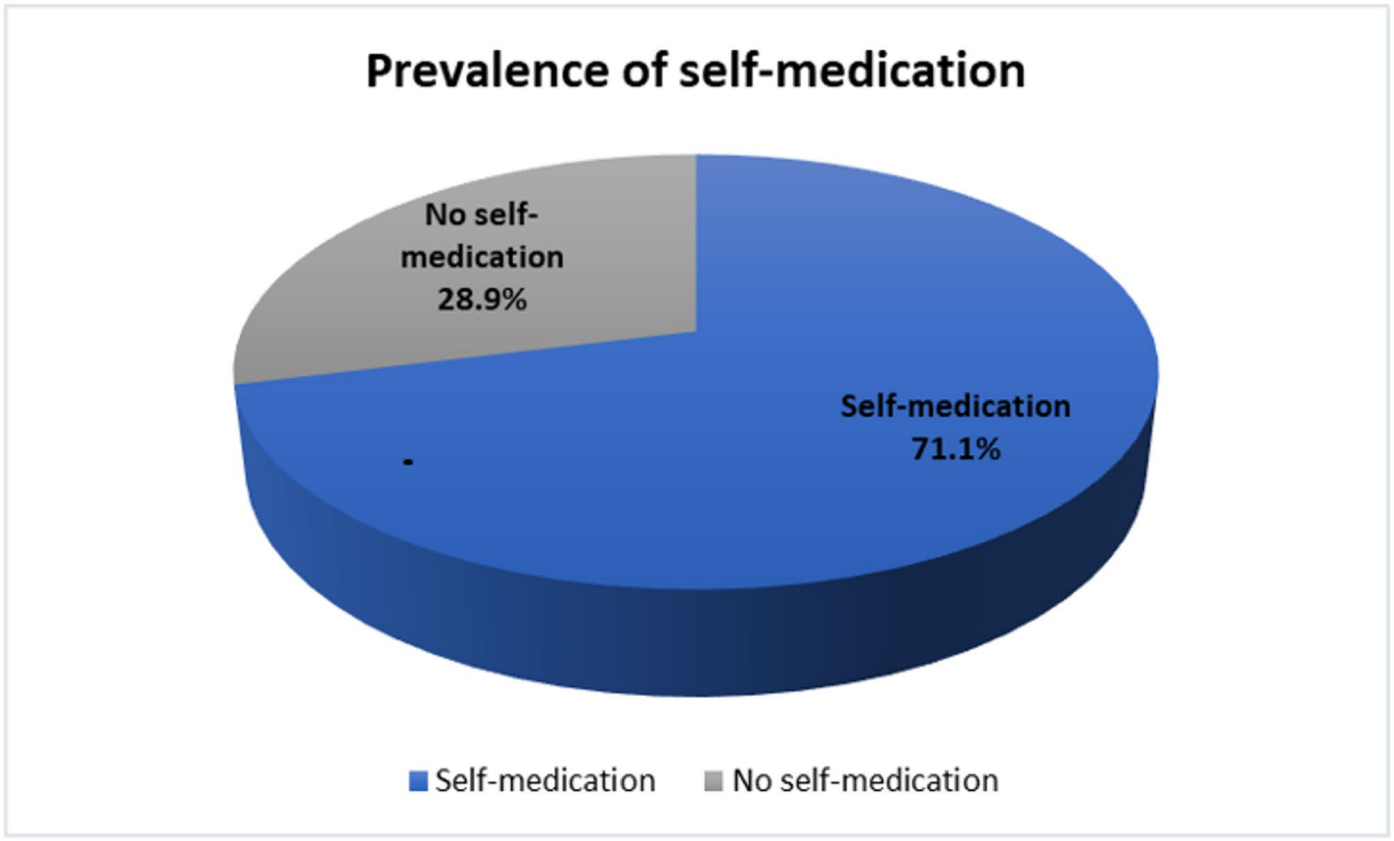
Prevalence of self-medication

The prevalence of SM was 71.1% (275/387). (Fig. 1)

### Reported outcomes of self-medication among respondents

**Fig. 2:**
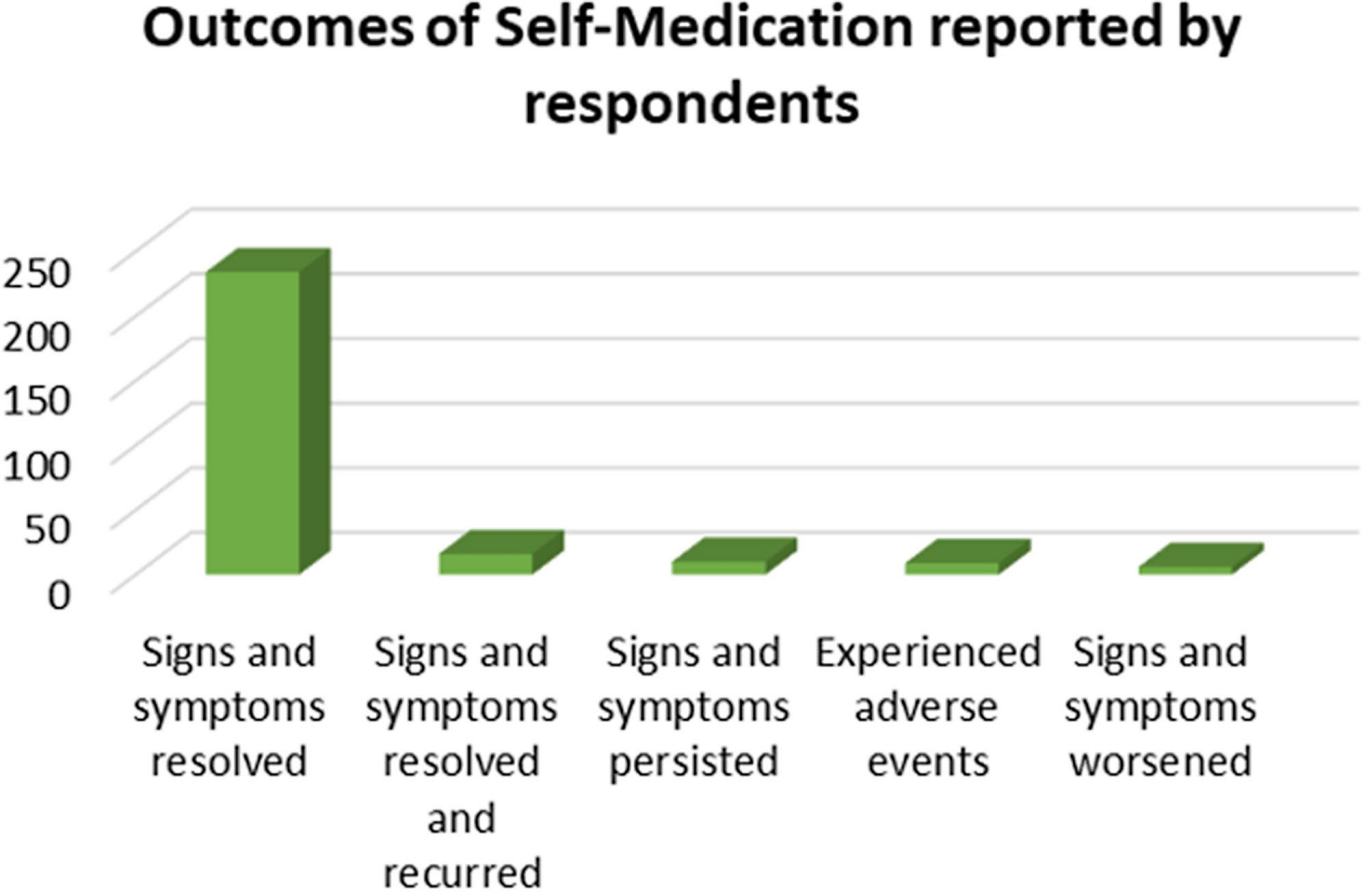
Reported outcomes of self-medication among respondents

Most of the respondents, 85.1% (234/275), who self-medicated reported resolution of signs and symptoms of their illnesses while 58.2% (16/275) had their signs and symptoms resolving and reoccurring after SM. Only 3.64% (10/275) of the respondents reported that their signs and symptoms persisted where as 3.27% (9/275) experienced adverse events (Fig. 2).

### Patterns of self-medication

#### Categories of medicines used for self-medication

**Fig. 3:**
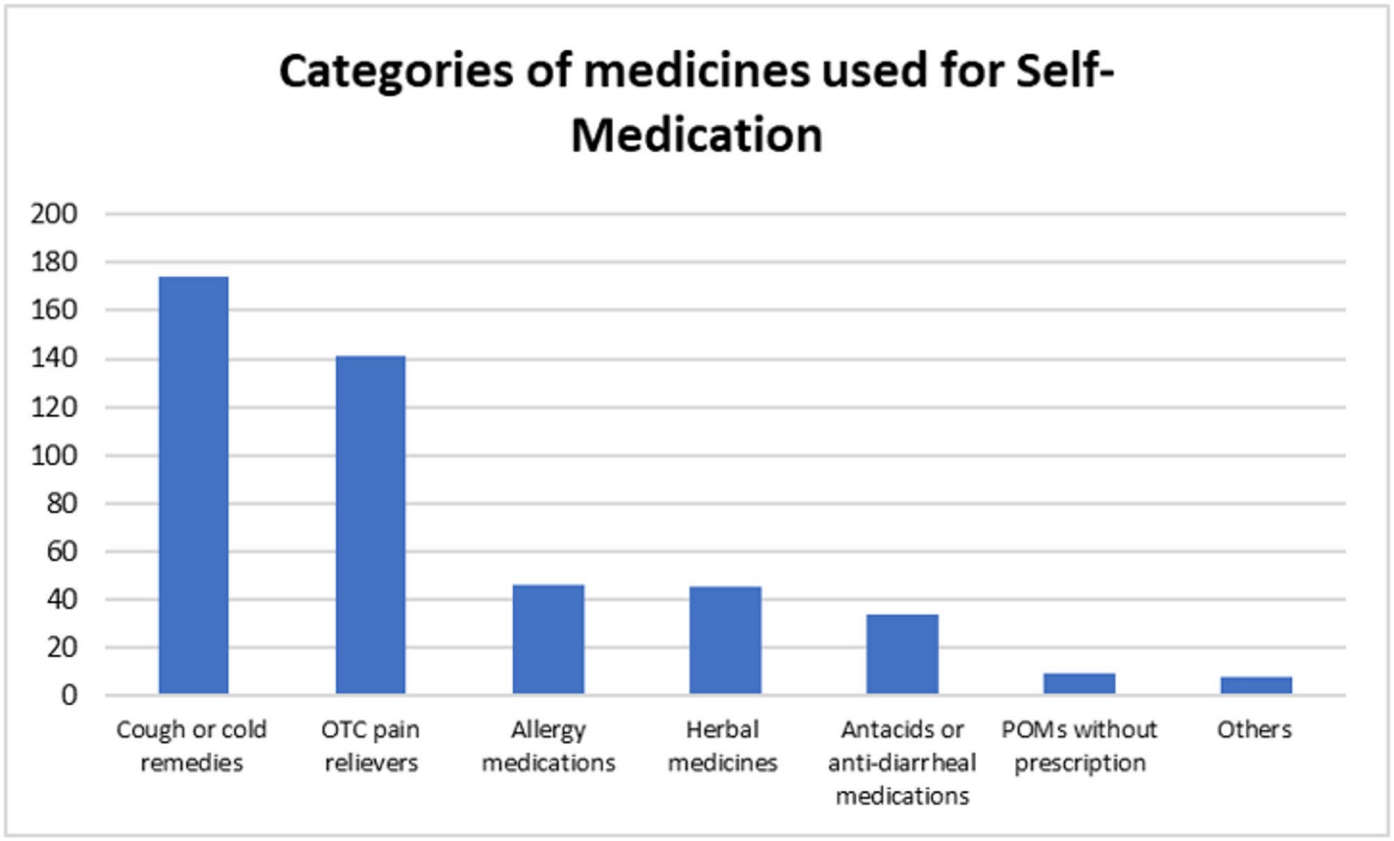
Categories of medicines used by respondents for Self-medication

The majority of the respondents who self-medicated used cough medicines; 63.3% (174/275), and OTC pain relievers; 51.3% (141/275). (Fig. 3)

### Signs and symptoms prompting respondents to self-medicate

**Fig. 4:**
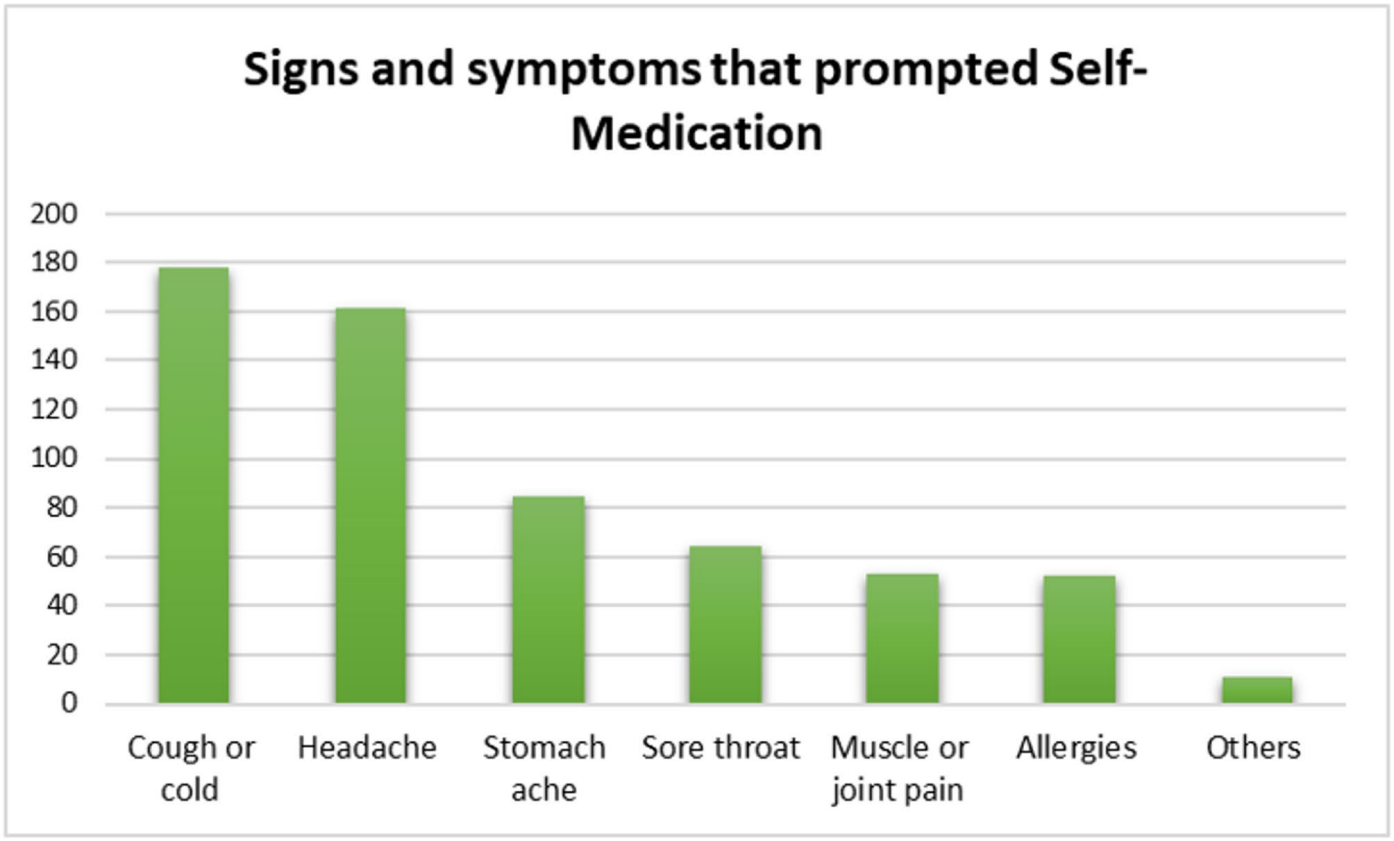
Signs and symptoms prompting respondents to self-medicate

The most common presenting complaints among students who were self-medicating were cough; 65.1%(179/275), and headache; 58.5%(161/275). (Fig. 4)

### Sources of medicines used by respondents for self-medication

**Fig. 5:**
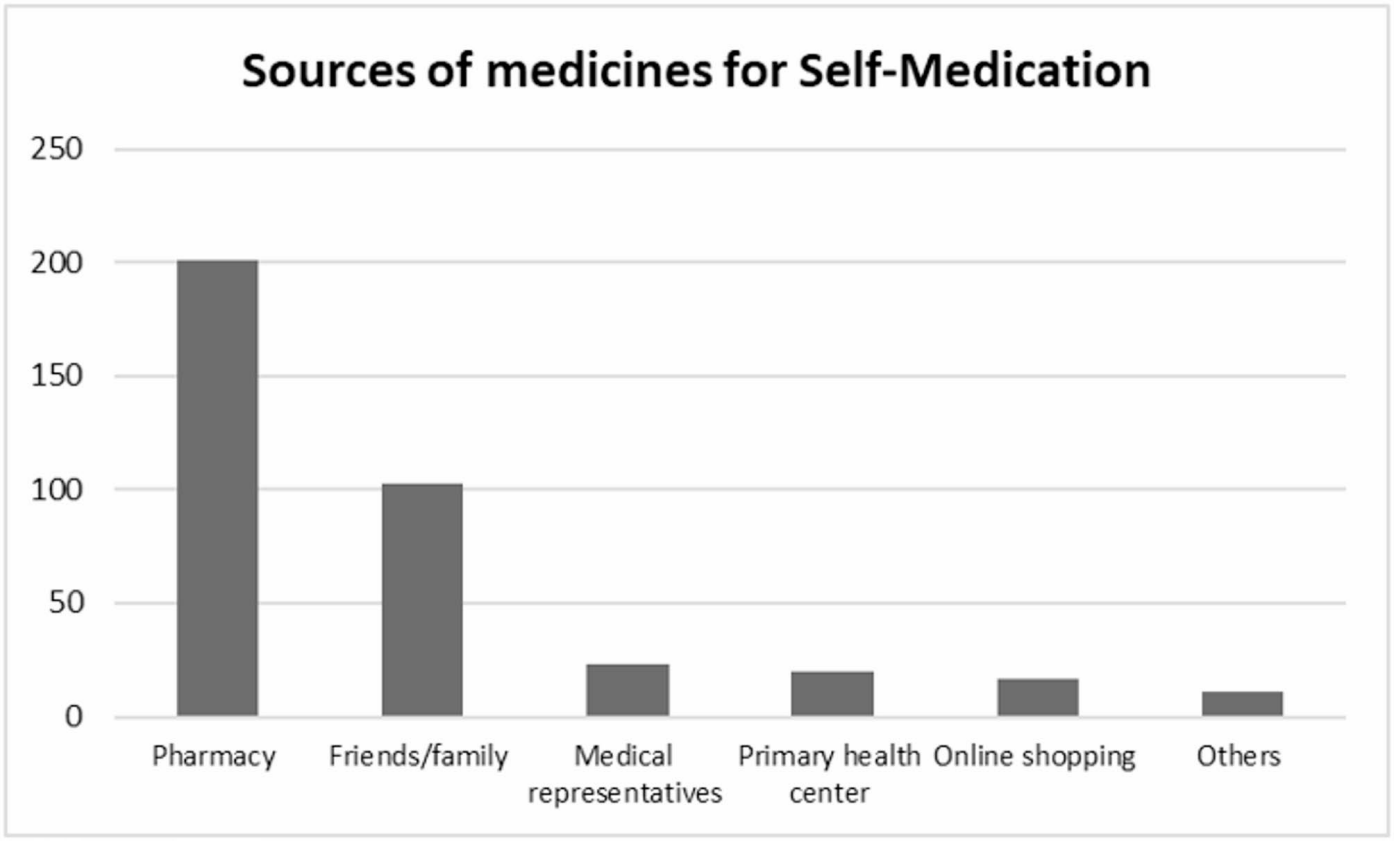
Sources of medicines used by respondents for Self-medication

Over half, 51.9% (201/387) of respondents, purchased drugs from pharmacies while 26.6%(103/387) obtained from friends/family. (Fig. 5)

### Sources of drug information prior to self-medication

**Fig. 6:**
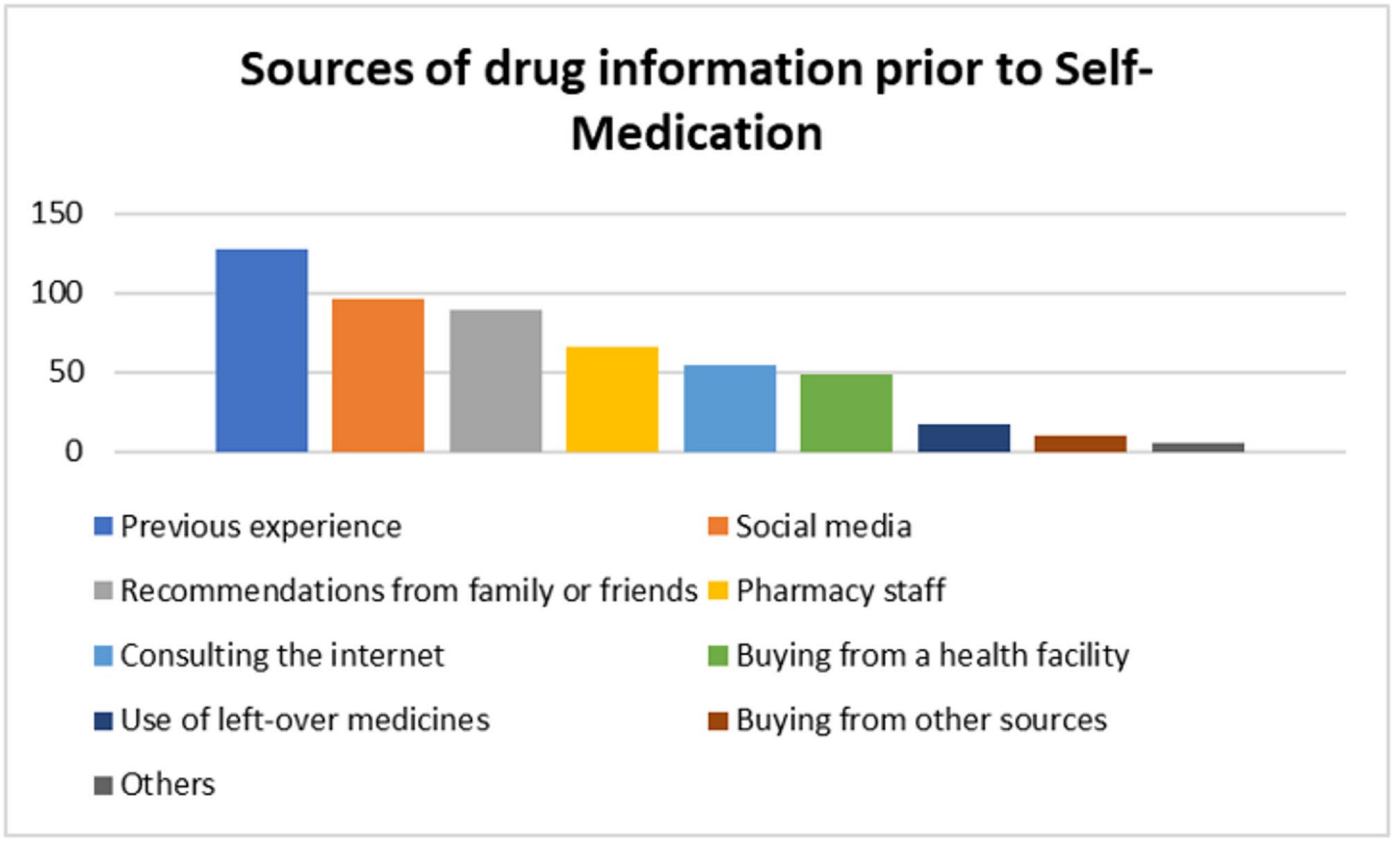
Respondents’ sources of drug information prior to Self-medication

Most respondents relied on knowledge from previous experience; 46.2% (127/275), social media; 34.9% (96/275), and recommendations from family/friends; 32.4% (89/275) to self-medicate. (Fig. 6)

### Respondents reasons for preferring self-medication

**Fig. 7:**
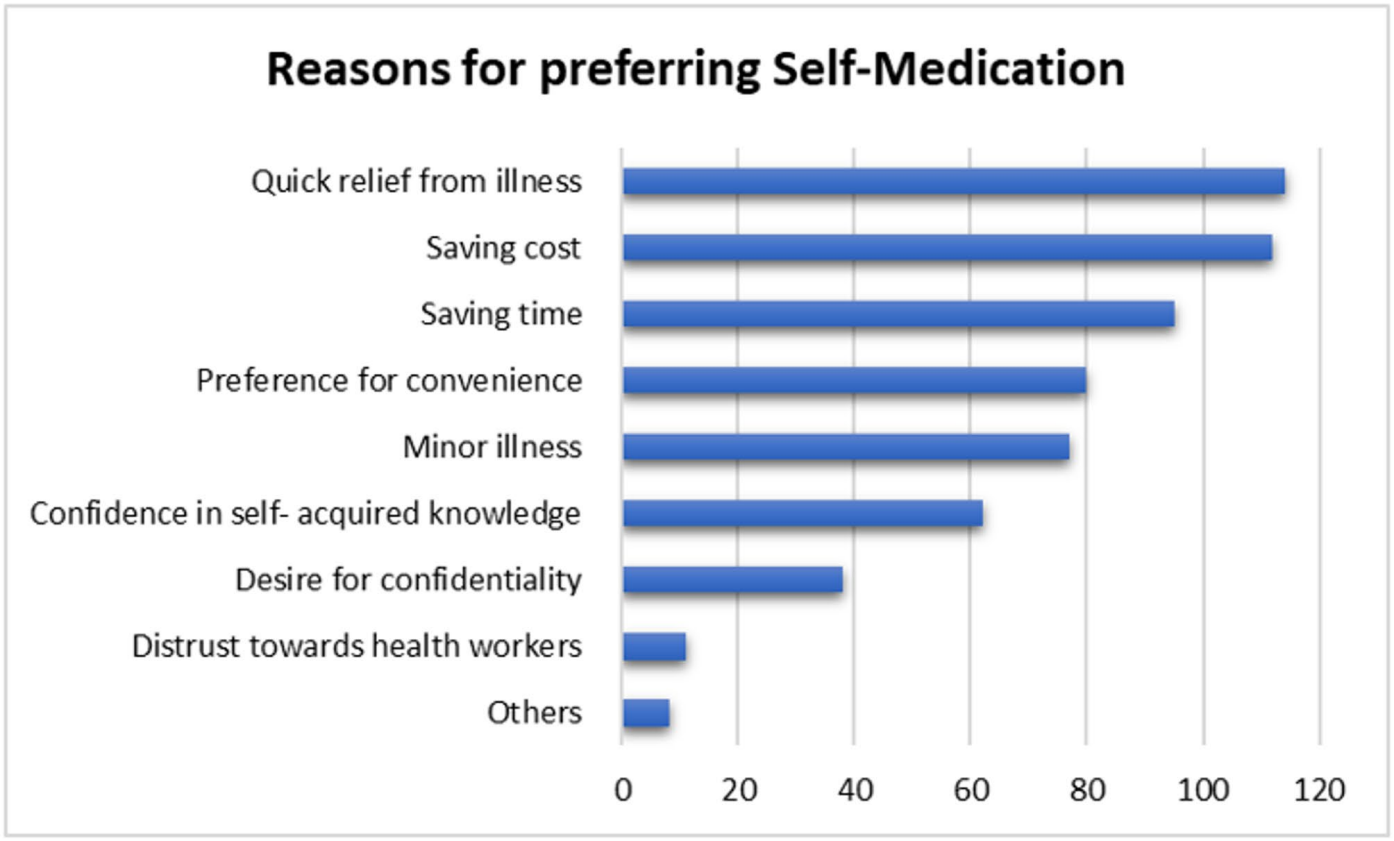
Respondents’ reasons for preferring Self-medication

Most respondents self-medicated to achieve quick relief from illness, 41.5% (114/275), save cost, 40.7% (112/275), and save time, 34.5% (95/275). (Fig. 7)

### Association between patterns of self-medication and resolution of signs and symptoms

Use of cough or cold remedies was significantly associated with resolution of signs and symptoms, X^2^(1, *N* = 173) = 5.668, *p* = 0.017. Among the various signs and symptoms that respondents presented with, having cough was observed to be significantly associated with resolution of signs and symptoms X^2^(1, *N* = 178) = 3.851, *p* = 0.050. Having a sore throat was also significantly associated with resolution of signs and symptoms X^2^(1, *N* = 64) = 4.983, *p* = 0.026. (Table 2)

**Table 2:** Association between patterns of self-medication and resolution of signs and symptoms

Patterns of self-medication	Resolution of signs and symptoms (Y = YES, *N* = NO)	
**Categories of medicines used for self-medication**	**Frequency (** ***n*** ** = 275)**	***p*** **-value**
Over-the-counter pain reliever (e.g. Paracetamol, ibuprofen) (*n* = 141)	Y (120) N (21)	0.994
Antacids or anti-diarrheal medicines (*n* = 34)	Y (27) N (7)	0.321
Cough or cold remedies (*n* = 173)	Y (154) N (19)	**0.017**
Allergy medications (e.g., antihistamines) (*n* = 46)	Y (38) N (8)	0.604
Prescription-only medicines not prescribed to you(*n* = 9)	Y (9) N (0)	0.202
Herbal medicines (*n* = 45)	Y (39) N (6)	0.746
Others (*n* = 8)	Y (8) N (0)	0.235
**Health conditions**		
Headache (*n* = 161)	Y (138) N (23)	0.730
Cough or cold (*n* = 178)	Y (157) N (21)	**0.050**
Sore throat (*n* = 64)	Y (60) N (4)	**0.026**
Muscle or joint pain (*n* = 43)	Y (38) N (5)	0.511
Stomach ache (*n* = 85)	Y (69) N (16)	0.223
Allergies (*n* = 52)	Y (40) N (12)	0.068
Others (*n* = 11)	Y (10) N (1)	0.580
**Drug source**		
Pharmacy (*n* = 201)	Y (171) N (30)	0.990
Primary health centre (*n* = 20)	Y (16) N (4)	0.507
Friends/family (103)	Y (92) N (11)	0.128
Online shopping (*n* = 17)	Y (17) N (0)	0.075
Medical representatives (*n* = 23)	Y (19) N (4)	0.727
Others (*n* = 11)	Y (9) N (2)	0.760
**Sources of drug information**		
Previous experience (*n* = 127)	Y (107) N (20)	0.717
Recommendations from family or friends (*n* = 89)	Y (80) N (9)	0.122
Social media (*n* = 96)	Y (82) N (14)	0.897
Pharmacy staff (*n* = 66)	Y (54) N (12)	0.392
Use of left-over medicines (*n* = 17)	Y (15) N (2)	0.707
Consulting the internet (*n* = 54)	Y (46) N (8)	0.983
Buying from a health facility (*n* = 48)	Y (40) N (8)	0.707
Buying from source other than health facility (*n* = 10)	Y (10) N (1)	0.580
Others (*n* = 5)	Y (5) N (0)	0.350
**Reasons for self-medication**		
Preference for convenience (*n* = 80)	Y (66) N (14)	0.440
Desire for confidentiality (*n* = 38)	Y (34) N (4)	0.409
Distrust towards health workers (*n* = 11)	Y (11) N (0)	0.157
Quick relief from illness (*n* = 114)	Y (97) N (17)	0.999
Confidence in self- acquired knowledge (*n* = 62)	Y (55) N (7)	0.363
Saving time (*n* = 95)	Y (79) N (16)	0.513
Saving cost (*n* = 112)	Y (89) N (23)	0.320
Minor illness (*n* = 77)	Y (64) N (13)	0.567
Others (*n* = 8)	Y (1) N (7)	< **0.050**

**Yes(Y)**: refers to number of respondents who reported resolution of signs and symptoms of their illnesses

**No(N)**: refers to number of respondents who did not report resolution of signs and symptoms of their illnesses

## Discussion

In the current study 71.1% of respondents had self-medicated over the past six months (Fig. 1). This is comparable with results of previous research done among students of Karachi university in Pakistan, where prevalence of SM was 76% [21]. In a systematic review and meta-analysis the national prevalence of SM in Uganda was 55.63% [22]. Unlike the current study which reports on a point prevalence the systematic review and meta-analysis included studies conducted between 1998 and 2024. This is could probably be the reason for the slight difference in the reported statistics. The relatively higher levels of education and drug knowledge among university students may have also influenced the comparatively higher prevalence of SM in universities [23]. The prevalence of SM reported in the current study was also comparable to findings from a previous study done among university students in Jordan which reported a prevalence of 69.3% [24]. The previous and current studies were conducted in LMICs which had comparable health challenges and constraints like understaffing, drug stock outs in public facilities, financial constraints within the communities, ineffective emergency response services. However, a higher prevalence of SM of 95.4% was observed in a study among university students in Nepal [25]. The study in Nepal enrolled a larger sample size of respondents compared to the current study and this could have created the observed difference in prevalences.

Majority of respondents in the current study reported resolution of signs and symptoms of their illnesses as an outcome of SM (Fig. 2). This observation was similar to one made among first-year medical students at Kampala International University [26]. This could have been due to increased access to online information courtesy of the university’s free Wi-Fi alongside drug reference applications like Medscape. Access to internet translates to access to information from various search engines and AI tools that may have been used by individuals for guidance when self-medicating. It also suggests that the respondents had a certain level of self-efficacy and resourcefulness, which probably had been impacted by prior experiences of self-care [27].

A point worth noting is some respondents in the current study were enrolled in medical programs. These had acquired some basic medical training. Due to their perceived knowledgeability about medicines and disease, they may have engaged in SM or could have prompted their peers to do the same [28]. This could have reinforced the practice of SM as observed in previous studies [21, 29, 30]. Previous studies have showed that SM is still wide spread among medical or pharmacy students despite having been exposed to knowledge regarding its dangers [21, 29]. Some of these students even perceived this practice as acceptable [30]. However, the patient’s condition may deteriorate in the absence of competent and prompt medical attention.

It is important to take into account that perceived knowledgeability among these students does not necessarily translate to competence nor expertise. A survey of students at an Iranian university found that pharmacy students knew more about drugs than their peers in other programs. Notwithstanding, the same study also reported a statistical significance in the correlation between the level of knowledge on drugs and the propensity for SM [31]. A similar observation was made in a systematic review where students pursuing medical programs were more likely to engage in SM compared to their non-medical counterparts [23]. These findings therefore denote that perceived knowledgeability is a driver of SM among university students pursuing health related programs.

In recent years, responsible SM has been considered an effective form of treatment for minor illnesses, especially in home management of mild symptoms of infections like COVID-19 or influenza [32]. It is a plausible argument that responsible SM may have some benefits like; the reduction in the cost of treatment, consultation time, and the workload of healthcare professionals [33, 34]. However, when all is said and done, all these convenient benefits and observed resolution of signs and symptoms after SM by no means imply safety from the dangers of SM. In any case it is reasonable to consider it the calm before the storm as risks like; drug addiction, adverse drug events, harmful drug-drug interactions are still apparent. On the other hand, it is also possible that the respondents in the current and previous studies experienced relief only because their illnesses were mild and self-limiting. These could have resolved by themselves without any contribution from SM [35].

Cough/cold remedies and OTC pain relievers were utilized by the majority of respondents (Fig. 3) to alleviate cough/cold and headache respectively (Fig. 4). Illnesses that are prevalent in LMICs such as respiratory tract infections, heart diseases, asthma, and the common cold usually present with a cough [36]. This could explain why cough was the commonest presenting complaint. Respondents also frequently took OTC pain medicines probably to alleviate discomfort associated with reduced quality of life [37]. Similar trends were also observed among university students in Saudi Arabia [38] and Sri Lanka [39]. The current and the previous studies were conducted in relatively low resource settings where cough and cold recur in the populations most likely because of the endemicity of respiratory infections [40]. This correlates with the fact that in the current study majority of the respondents self-medicated based on previous experience.

Most respondents obtained their medicines from pharmacies, friends and family members (Fig. 5). This result is consistent with findings from a prior study conducted among students at MUST, the same setting in which the current study was conducted [12]. This could have been due to ease, accessibility, or perceived safety experienced by respondents visiting community pharmacies [41]. The proportion of respondents who obtained remedies from friends or relatives was somewhat higher in the current study compared to the previous study at MUST. This demonstrates a rising trend in this trait that could be explained by the popularity of social media platforms that families and friends use for communication and networking. The observed inclination to get medicines from friends or family may have been a result of the desire among the youth to network and socialize.

Social media as a means of communication is tremendously popular among university students and no wonder it ranked only second among the most used sources of drug information among respondents (Fig. 6) [42]. The dependence on advice from friends and family highlights how social networks influence SM behaviours. Although the community may view such recommendations as reliable, they are rarely grounded in evidence-based literature. It implies that greater awareness-raising regarding formal healthcare is necessary [43]. Due to the fact that some of the social acquaintances may have had some medical training, they may have offered convenient recommendations on disease and medicines leading some respondents to self-medicate [44].

The desire for immediate relief from illnesses, evading expenses associated with consulting a licensed prescriber, saving time and preference for convenience were the main reasons for SM in the current study (Fig. 7). A study done to investigate perspectives of Iranian nursing students on SM showed that students in tertiary institutions resorted to this trait because it was presumably perceived to save time and to be more convenient as opposed to consulting a licensed prescriber [45]. Some of these findings were similarly observed in studies conducted in Bosomtwe District, Ghana [46] and in the health science colleges of Majmaah University in Saudi Arabia [47].

A statistically significant correlation was observed between resolution of signs and symptoms and; occurrence of cough, sore throat and use of cough or cold remedies (Table 2). Cough, cold and sore throat are commonly caused by respiratory tract infections [48, 49]. Respiratory infections are endemic in sub–Saharan Africa therefore communities in this setting may be familiar with management of these ailments due to repeated exposure [50–52]. Previous studies have also demonstrated that respiratory tract infections are common among students attending tertiary institutions which has similarly been observed in the current study [31, 53–55]. Students who are pursuing medical programs for which clinical training in hospital wards is a requirement are by default exposed to patients with respiratory infections which they might catch and spread to their colleagues.

Headaches, fevers and pains have been observed to be common reasons for SM in previous studies [56–59]. However, in the current study these did not demonstrate a significant statistical association with resolution of signs and symptoms. Headache, fever and pain are indicators of underlying illness. Despite adequate treatment of these signs and symptoms the underlying pathology needs to be adequately treated. Otherwise, the same signs and symptoms may recur or persist. It is noteworthy that even in previous studies there is no demonstration of statistical significance in the association of between headache, fever, pain and resolution of signs and symptoms.

## Conclusion

The findings of the current study are suggestive of the popularity of SM among students attending university in sub-Saharan Africa. This study also brings to light the significance of SM to the health care of university students. However, it is key to note that resolution of signs and symptoms after SM does not necessarily translate to safety from its harmful consequences. A statistically significant correlation was observed between resolution of signs and symptoms and; occurrence of cough, sore throat and use of cough/cold remedies. It is noteworthy that the seemingly convenient outcomes observed in the current study must not be considered as justification to always substitute conventional medical consultations with SM. Therefore, there is enormous need to not only educate communities on the dangers of SM but also on how it can be practiced responsibly.

## Electronic supplementary material

Below is the link to the electronic supplementary material.

Supplementary Material 1

## Data Availability

The data that support the findings of this study are not openly available due to reasons of sensitivity and are available from the corresponding author upon reasonable request.

## Abbreviations

HIC: High-income country
KIU: Kampala international university
LMIC: Low- and middle-income country
MUST: Mbarara university of science and technology
NCHE: National council for higher education
OTC: Over the counter
POM: Prescription only medicine
SM: Self-medication
SPSS: Statistical package for the social sciences
WHO: World health organization
