# Supplementary material for: Resolution of signs and symptoms of illnesses among self-medicating undergraduate students of Mbarara University of Science and Technology: a cross-sectional study
Source: J Pharm Health Care Sci. 2025 Jul 25;11:62. doi: 10.1186/s40780-025-00469-8 (PMC12291416; doi:10.1186/s40780-025-00469-8)
Supplement: Supplementary file 1 — Supplementary Material 1 [file 40780_2025_469_MOESM1_ESM.pdf]

QUESTIONNAIRE

Serial number: ____

Study topic: Patterns and Outcomes of Self-medication among Undergraduate Students Enrolled at Mbarara University of Science and Technology: A Cross-sectional study.

Please answer the following questions by crossing (×) against one of the options provided. Your answer will be kept confidential and will only be used for research purposes.

Sociodemographic

1. Gender: ☐ male ☐female ☐do not wish to specify
2. Age:

   ☐18-20

   ☐21-23

   ☐24-26

   ☐27 or above
3. Marital status:

☐Married	☐single
☐Divorced	☐
☐Widowed	

4. Residence: ☐University halls ☐Hostels/rentals ☐Commute from home
5. Religion: ☐ Catholic ☐Anglican/protestant ☐Muslim ☐Pentecostal ☐ Others
6. Academic sponsorship:

Government scholarship ☐ private scholarship☐

7. Faculty:

☐Faculty of medicine

☐Faculty of Interdisciplinary Studies

☐Faculty of business and management sciences

☐Faculty of applied science and technology

☐Faculty of computing and informatics

☐Faculty of Science

8. Year of study:

☐Year 1

☐Year 2

☐Year 3

☐Year 4

☐Year 5

**Self-medication patterns**

Definition of self-medication:

Self-medication is the selection and use of medicines by individuals to treat self-recognized illnesses or symptoms.

9. Have you self-medicated in the last six months?

   ☐Yes

   ☐No, if no, then stop here.

10. If yes, please specify the categories of medications you have self-medicated with (check all that apply):

    ☐over-the-counter pain reliever (e.g. Paracetamol, ibuprofen)

    ☐Antacids or anti-diarrheal medications

    ☐Cough or cold remedies

    ☐Allergy medications (e.g. antihistamines)

    ☐Prescription-only medications not prescribed to you

    ☐Herbal medicines

    ☐Others ………….

    NOTE;

    Herbal medicines mean any finished labeled medicinal product that contains active ingredients of aerial or underground parts of plants or other plant material or a combination of them, whether in a crude state or as plant preparation.

    What health conditions or symptoms have you self-medicated for? (check all that apply)

    ☐Headache

☐Cough

☐Sore throat

☐Muscle or joint pain

☐Stomach ache

☐Allergies

☐Other…………….

11. Where do you obtain your drugs for self-medication?

You can select more than one option if obtained from more than one source

☐Pharmacy	☐Online shopping
☐Primary health center	☐Medical representatives
☐Friends/family	Others☐

12. How do you usually obtain information about the medications you use for self-medication? (check all that apply):

☐Previous experience

☐Recommendations from family or friends

☐Social media

☐Pharmacy staff

☐Use of left-over medicines

☐Consulting the internet

☐ From a health facility

☐ Advertisements

☐Others………………

13. What are the main reasons why you choose to self-medicate? (check all that apply)

☐ Preference for convenience

☐Desire for confidentiality

☐Distrust towards health workers

☐Quick relief from illness

☐Confidence in self-acquired knowledge

☐Saving time

☐Saving cost

☐Minor illness

☐Other ……………….

**Outcomes of self-medication**

14. What were the outcomes of your self-medicating?

☐Symptoms and signs resolved (Cessation of signs and symptoms)

☐Symptoms and signs worsened

☐The patient experienced adverse drug events. **If yes, continue to question 15**.

☐Symptoms and signs persisted

☐Symptoms and signs resolved and reoccurred after self-medication

15. What did you do about the adverse events you experienced?

☐Go to the private sector	☐Go to pharmacist
☐Go to primary health care center	☐Stop taking medication
☐Increase dosage	☐Decrease dosage
☐Switch to another drug	☐Others
